# Bringing next‐generation diagnostics to the clinic through synthetic biology

**DOI:** 10.15252/emmm.201606541

**Published:** 2016-07-11

**Authors:** Alexis Courbet, Eric Renard, Franck Molina

**Affiliations:** ^1^ Sys2diag FRE3690 CNRS/ALCEDIAGMontpellierFrance; ^2^ Department of Endocrinology, Diabetes, Nutrition and INSERM 1411 Clinical Investigation CenterUniversity Hospital of MontpellierMontpellier Cedex 5France; ^3^ Department of BiochemistryUniversity of WashingtonSeattleWAUSA; ^4^ Institute for Protein DesignUniversity of WashingtonSeattleWAUSA; ^5^ Institute of Functional GenomicsCNRS UMR 5203INSERM U1191University of MontpellierMontpellier Cedex 5France

**Keywords:** Systems Medicine

## Abstract

The promise for real precision medicine is contingent on innovative technological solutions to diagnosis and therapy. In the post‐genomic era, rational and systematic approaches to biological design could provide new ways to dynamically probe, monitor, and interface human pathophysiology. Emerging as a mature field increasingly transitioning to the clinics, synthetic biology integrates engineering principles to build sensors, control circuits, and actuators within the biological substrate according to clinical specifications. A particularly tantalizing goal is to develop novel versatile, programmable and autonomous diagnostic devices intertwined with therapy and personalized for the patient to get closest, finest, and most comprehensive diagnostic information and medical procedures. Here, we discuss how synthetic biology could be preparing the future of medicine, supporting and speeding up the development of diagnostics with novel capabilities to bring direct improvement from the clinical laboratory to the patient, while addressing healthcare evolution and global health concerns.

## Perspectives on diagnostic technologies

Developing high clinical value diagnostics remains a major technological challenge of medical sciences. Conventional diagnostic technologies are facing evolving economical and resource imperatives, while still mostly relying on analytical chemistry or antibody‐based platforms centralized in clinical laboratories to match standards of specificity, sensitivity, throughput, and robustness. However, these approaches are often not clinically informative enough to accommodate precision medicine, and confront complex syndromes, rare or rapidly emerging diseases.

Obtaining a comprehensive view of patient pathophysiology, its exposure to the environment and its trajectory over time require new modes of detection for complex and dynamic biomarker signatures still mostly inaccessible to measurement. For instance, most microbiological diagnostics cannot provide timely infectious etiology before tedious laboratory manipulations, thereby imposing important mortality burdens. Likewise, achieving dynamic, real‐time, and non‐invasive monitoring of endocrine function, exposure to toxics, metabolic parameters such as glycemia, or early molecular onset of cancer would constitute medical breakthroughs.

This would require autonomous diagnostics capable of direct analysis of new types of biological parameters in complex matrices. Ultimately supported by portable and low‐cost devices, implantable diagnostics could prove extremely valuable in many clinical situations that would benefit from precise processing of localized cellular and molecular information. Interestingly, integration of medical reasoning within diagnostics in the form of programmable signal processing could allow complex measurements while considerably speeding up and improving diagnostic to treatment efficiency. Engineering the next‐generation diagnostic devices to meet modern medical needs remains a critical challenge, which necessitates innovative approaches and is therefore increasingly drawing the interest of synthetic biologists.

## Synthetic biology: a transformative medical biotechnology

**Figure 1:**
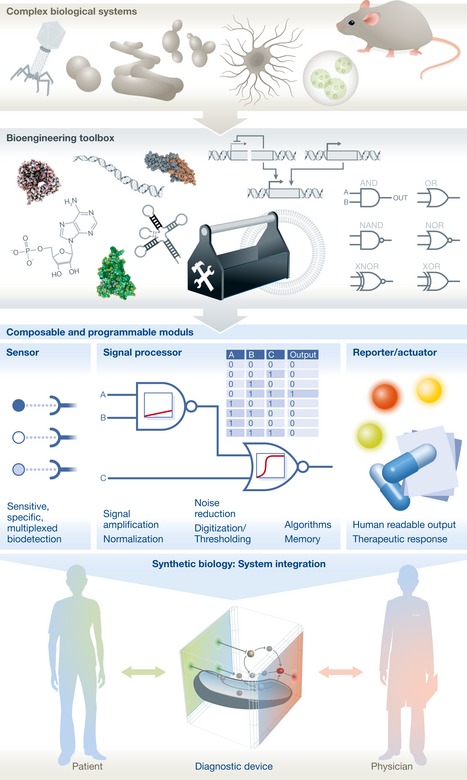
Synthetic biology enables the engineering of next‐generation diagnostics through the system integration of sophisticated biological capabilities Biological systems have evolved powerful molecular modules to sense and process biological signals and inform their phenotypes accordingly. Synthetic biology enables the systematic re‐engineering, standardization, and cataloguing of useful biological parts. It supports hierarchical abstraction from biological complexity for efficient assembly of parts into useful, composable, and programmable modules. Medically relevant modules for sensing biomarkers, achieving signal processing, and reporting can then be further integrated into biosensing systems to develop novel diagnostic devices meeting clinical specifications to aid medical decision‐making.

The last decades can be regarded as the descriptive phase of molecular biology that permitted the advent of synthetic biology. Synthetic biology has become a science of structuring biological matter in a rational and systematic manner to achieve control on energy and information processing through classical engineering strategies: standardization, hierarchical abstraction of complexity, and modularity (Way *et al*, 2014). It provides a methodology to exploit the vast repertoire of biomolecular components generated by evolution, ultimately contemplating the design of orthogonal biological systems on purpose (Fig 1). Standard biological parts are catalogued in accessible registries based on quantitative data, enabling the decoupling of biotechnological fabrication from design. This in turns lowers the cost, shortens the development to production process, and increases the scale of biological design while integrating robustness and reliability specifications.

Through the prism of information technologies, it has prompted the engineering of biological control circuits such as synthetic gene and biochemical networks to program specific sequences of operation *in vivo* or *in vitro*. This was illustrated in the early success of various genetic modules: sensors, switches, oscillators, counters, cell–cell communication, or biomolecular Boolean logic. These advances are constantly augmented in terms of scale with, for instance, automated design of analog/digital information processing and storage systems or metabolic pathways toward bioproduction of synthetic biomaterials, biofuels, and biomolecules.

Recently, considerable advances have permitted the growth of medical synthetic biology. Most remarkable example of success concern vaccine production, synthesis strategies for high‐value drugs such as artemisinin, synthetic opioids, or novel antibiotics, as well as medical biomaterials, gene delivery tools, control of parasite vectors, or a vast range of proof‐of‐concept therapeutic smart cells.

## How can synthetic biology be useful to medical diagnosis?

**Figure 2:**
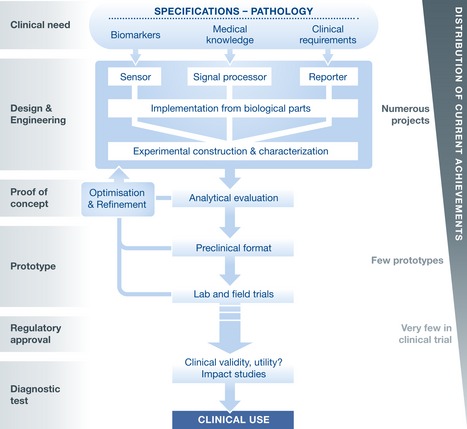
Systematic workflow for the development of diagnostic devices using synthetic biology, from bioengineering considerations to clinical use A bioengineering solution can be formulated corresponding to certain clinical specifications as a technological support to disease diagnosis. Such specifications can be systematically implemented within biological substrate using standardized biological parts. An iterative process between analytical properties of engineered diagnostic systems, design, and construction optimizes the process to eventually lead to effective approval and clinical use.

Living systems are problem‐solving biomolecular machines that perform ultrasensitive and specific responses to a vast range of biochemical signals. They are autonomous and self‐organizing, and can function in complex biological contexts at all scales. Synthetic biology enables the rational engineering of novel diagnostic biosensing systems with a modular architecture consisting of sensor, processor, and reporter modules (Fig 1). Sensed molecular events can be associated with specific signal processing operations supported by synthetic circuits, which can integrate medical knowledge to classify patient conditions into clinical categories. The processor module can be programmed for multiplexed detection of pathological signals according to various medical decision rules. Various modalities of measurements can also be integrated: quantitative, semiquantitative or qualitative, pathological signals can be amplified, noise‐filtered, or thresholded. Such a modular and standardized interface between sensor and reporter components can speed up the design while increasing its versatility. Moreover, synthetic systems can be systematically and rapidly programmed to integrate varying clinical constraints, emergent pathologies, and disease heterogeneity and complexity. Synthetic biology thus allows for an unprecedented manipulation of the biological substrate for tailored signal detection, processing, and reporting, to the full integration into autonomous devices evaluating diagnostic rules *in situ*, either *in vitro* or *in vivo* (Fig 2).

## What has synthetic biology proven so far in the field of diagnostics?

*In vitro* assays performance can be improved via synthesis of standardized and robust diagnostic reagents. Synthetic multifunctional antibodies, synthetic genes and oligonucleotides, or multi‐epitope and chimeric antigens can reduce assay variability and achieve high levels of sensitivity and specificity. Novel diagnostic targets can be accessed, for instance, providing with immunoassays of autoimmune diseases or neurological syndromes, as well as DNA arrays for newly described pathogens. Integration of abiotic synthetic biology on paper formats recently brought considerable attention. Freeze‐dried synthetic gene circuits can be programmed for the diagnostic of various pathologies and integrated on paper devices to provide with extremely low cost and fast development, high versatility, portability, and scalability. Proofs of concept demonstrated colorimetric detection of small molecules and nucleic acids, such as glucose, bacterial antibiotic resistance genes, and strain‐specific Ebola or Zika virus diagnostics (Pardee *et al*, 2014), which highlight capabilities as rapid programmable biosensors to tackle global epidemics. Using the same approach, point‐of‐care companion diagnostics relying on synthetic proteins showed capable of precise quantification of narrow therapeutic range immunosuppressant drugs in drops of serum (Griss *et al*, 2014).

Complex biomolecular machines can also be engineered into novel diagnostics. For instance, reengineering the ability of bacteriophages to specifically infect and lyse bacterial hosts provided with near‐real‐time pathogen diagnostics capable of antibiotic susceptibility testing in raw clinical samples, potentially revolutionizing clinical microbiology where well‐recognized limitations arise from long and tedious cultivation phases. High‐throughput methods relying on bacteriophages also permitted to explore a patient's serological repertoire for all human viruses at a time from a single drop of blood (Xu *et al*, 2015). Versatility of bacteriophage diagnostics could dramatically improve the detection of human pathogens for industrial and clinical applications (Citorik *et al*, 2014).

Synthetic biologists have also long been developing living cell biosensors. Easy and inexpensive to engineer and produce, they require low‐cost reagents and have evolved increased robustness to perturbation. Cell‐based biosensors can provide functional and physiological information compared to classical analytical methods. Because of the self‐replication of biological systems, self‐powering and resistance forms, bacterial, yeast, or mammalian diagnostic cells can be integrated into portable devices compatible with widespread deployment. We recently engineered programmable bacterial biosensors embedding synthetic digital gene circuits (Courbet *et al*, 2015). Insulated into disposable, handleable, and portable format, they achieved multiplexed diabetes‐associated biomarker detection in raw clinical samples while integrating programmable medical decision algorithms and long‐term memory. Similarly, mammalian designer cells embedding synthetic G‐protein signal processing module allowed precise and personalized profiling of allergies in human whole‐blood samples with clinical sensitivity and dynamic ranges (Ausländer *et al*, 2014). This strategy proved extremely interesting when current *in vivo* and *in vitro* diagnostic methods to determine the molecular etiology of allergic syndromes suffer from lack of reproducibility, patient discomfort, bulky experimentation, and poor correlation with clinical symptoms.

Synthetic cell‐based biosensors also hold promises as autonomous devices for continuous monitoring of pathophysiology *in vivo*. For instance, human commensal bacteria can be engineered with sensor, control circuits, memory, and reporters to score molecular events and deliver diagnostic information non‐invasively. They could be deployed for the monitoring of intestinal disorders, pathogens, inflammatory state, cancer, or microbiome dysbiosis. For instance, engineered probiotics have been shown capable of selectively targeting tumoral microenvironments in the gut, detecting the onset of hepatic cancerogenesis and generating high‐contrast signal in the urines of live animals (Danino *et al*, 2015). Likewise, designer mammalian cells have the potential to yield *in vivo,* implantable diagnostics of particular interest. Genetically encoded in living cells, diagnostics can be coupled with therapeutics *in situ* (i.e. theranostics). This strategy involves synthetic gene circuits acting as intracellular molecular prosthesis that autonomously monitor disease‐associated biomarkers and coordinate adjusted and timely therapeutic responses. This approach could prove extremely valuable in many diseases with asymptomatic onset, complex dynamics, and clinical situations where therapeutic benefit depends on the delay in analytical methods, information management, interpretations, and effective patient care, for instance, in the long‐term surveillance of cancer, or chronic autoimmune, infectious and inflammatory diseases. For example, mammalian cells were engineered to detect clinical levels of psoriasis‐specific TNF and IL‐22 cytokines and deliver systemically IL‐4 and IL‐10 immunomodulatory cytokines in real time. When implanted *in vivo,* these devices prevented psoriatic onset, stopped acute events, and restored physiological tissue morphology. Similarly, engineered autologous T cells have been tested in clinical trials for cancer theranostics, and a variety of adoptive cell therapeutics approaches could benefit from constantly increasing precision and capabilities. Mammalian cells have also been designed to perform metabolic disorders, urate homeostasis, obesity, and diabetic ketoacidosis management (Kojima *et al*, 2016). Further, intracellular diagnostics can be engineered to integrate programmable logic and sensing to classify complex pathogenic patterns of cancer‐related gene expression or miRNAs *in vivo* triggering selective and robust apoptotic responses to malignancy. Using gene expression levels that are commonly used to diagnose prostate and small‐cell lung cancer, synthetic genes circuits could be programmed to multiplex molecular biomarkers *in vivo* and compute appropriate therapeutic outputs. Alternatively, devices made of networked synthetic nucleic acids have been developed, such as autonomous DNA nanomachines capable of multiplexed analysis of cancer cell surface markers and logic‐gated delivery of medical molecular payloads in live animals.

Since the lack of specific and predictive endogenous biomarkers limits the diagnosis of many complex diseases, attention has recently been given to the engineering of synthetic biomarkers. Administered in the circulatory system to record molecular events associated with pathological states, they could enable the non‐invasive monitoring of non‐classical parameters by producing disease‐specific molecular signatures. The potential for early disease stage detection and monitoring has been reported with experimental models of liver fibrosis, cancer and solid tumors, or cardiovascular diseases. For instance, synthetic biomarkers scoring pathological enzymatic activities at disease sites release reporters in circulation that concentrate in urines to be measured user paper devices (Warren *et al*, 2014).

## Concluding remarks

Synthetic biology has advanced enormously in the past few years. Engineered molecular and cellular devices with biosensing and information‐processing capabilities proved to be clinically compliant and are now transitioning to trials. It provides with an opportunity to decrease the size and resource requirements of diagnostic devices, increase portability to get closer to the patient, and give access to real‐time, personalized, and physiologically meaningful diagnostic measurements (Table 1). The capacity to satisfy point‐of‐care and companion diagnostics could bring these evolutions to the patient as well as into the clinical laboratory. Further, the prospect for *in vivo* synthetic devices to act as self‐contained theranostics is almost established and could evolve toward multipurpose implantable systems, potentially introducing a new era for clinical practices and precision medicine. Realizing these novel approaches could benefit individuals as well as society, improving therapeutic outcome and reducing healthcare costs, while also benefiting regions with poor infrastructure.

**Table 1:** Conceptual differences in medical procedures between conventional versus synthetic biology enabled diagnostics

	Conventional diagnostics	Synthetic biology enabled diagnostics
Diagnostic procedure	Centralized clinical biochemistry laboratory, high resource requirements	Ambulatory, close to patient, potentially *in vivo,* low resource, delocalized
Sample management	Pre‐treatment, large volumes	Raw, small volumes
Nature of biomarkers	Parallelized, static, disconnected from patient pathophysiology	Multiplexed, dynamic, *in situ*, close to patient pathophysiology
Data transmission	Delayed, complex interfaces	Real‐time, integrated signal processing, local/remote readout
Link to therapy	Delayed, through physician evaluation	Direct, *in situ*, through programmable decision algorithms: remote supervision
Data management	Files, registries	Embedded memory
Medical benefit	Robustness, gold standard	Patient comfort/care, personalized solutions, patient commitment
Development	High cost and lengthy	Short and low‐cost design to production

Synthetic biology could serve as a technological support for medical biology, simplifying decision rules for clinicians through more precise and sophisticated diagnostic tools. However, uncertainty remains about the extent to which clinicians will to adopt these diagnostic tools. Although recent technological biosafety and risk assessment advances have been made, regulatory concerns remain to be addressed. In this perspective, abiotic synthetic biology is likely to provide the next medical tools and paper‐based diagnostic platforms appear extremely promising.

Last, we envision that the field also holds considerable value to explore human pathophysiology. The reverse engineering of biosynthetic pathways, genes, and networks constitutes resources for the multi‐level screening of disease mechanisms. It allows iterative design and *in vivo* implementation of quantitative models to test molecular hypotheses and probe biological networks. In that perspective, synthetic biology represents an unprecedented approach to exploring pathophenotypes, discovering new biomarkers and augmenting molecular definition of syndromes.

## Conflict of interest

The authors declare that they have no conflict of interest.
